# Effects of Temporal Expectancy on Motor Control and Cognitive Processes Supporting Speech Production

**DOI:** 10.3390/brainsci16080817

**Published:** 2026-07-31

**Authors:** Jayanthi Sasisekaran, Benjamin Eisenreich, Juergen Konczak

**Affiliations:** 1 Department of Speech-Language-Hearing Sciences, University of Minnesota, Minneapolis, MN 55455, USA; 2 Center for Translational Sensory Sciences, University of Minnesota, Minneapolis, MN 55455, USA; beisenre@umn.edu; 3 Department of Kinesiology, University of Minnesota, Minneapolis, MN 55455, USA; jkonczak@umn.edu

**Keywords:** temporal predictability, speech motor, event-related potentials

## Abstract

**Highlights:**

**What are the main findings?**

- Temporal predictability facilitated speech motor planning and engaged endogenous attention.
- Speech onset was influenced by the cognitive demands of the task and differed between trials requiring overt speaking vs. response switching.

**What are the implications of the main findings?**

- Temporal predictability primes speech motor planning and enables faster speech.
- Response switching imposes cognitive and motoric effort, delaying speech onset.

**Abstract:**

**Background/Objectives**: This study investigated the effects of temporal predictability on electrocortical processes underlying speech motor control and cognition. **Methods**: Twenty-one adults completed an S1–S2 (prompt for speech) visually cued delayed speech production task. The task required saying noun phrases or switching to saying the noun at 1.5 s vs. 3 s inter-stimulus intervals (ISI). Event-related potential indexing attentional effort (Contingent Negative Variation, CNV), speech motor planning (Bereitschaftspotential), speech programming (Late Readiness Potential, LRP), and speech onset latencies were compared between the categories using mixed effects analysis. **Results**: At 400–600 ms before S2 cue onset and speech motor planning, the 1.5 s ISI condition showed significantly higher mean amplitude of CNV at bilateral anterior frontal, central, and parietal electrode sites. At 400–600 ms, the Bereitschaftspotential representing movement planning showed a larger mean amplitude in the 1.5 s ISI condition at anterior frontal and central electrode sites that was moderated by CNV as covariate. Significant differences were evident between the speak and response switch conditions in LRP and speech onset. **Conclusions**: ISI-based effects of temporal predictability on speech motor planning, speech onset, and the cognitive processes supporting speech production are discussed.

## 1. Introduction

Speech is considered a complex motor act involving the planning and coordination of several muscle synergies at the respiratory, laryngeal, and articulatory levels. Several factors, including temporal predictability and task complexity, are known to influence motor control processes. Cognitive processes also play a significant role in speech production. The aim of this study is to examine temporal expectancy as a factor influencing the speech motor and cognitive processes underlying speech production.

### 1.1. Models of Motor Control and Temporal Predictability

Models of speech motor control, including the Directions into Velocities of Articulators (DIVA [1,2]) and the State Feedback Model (SFM [3]), postulate a feedforward mechanistic account of speech production. The DIVA model postulates that when a motor plan is initiated, an efference copy or corollary of the expected sensory consequences is shared with the auditory and somatosensory cortices. Similarly, internal state feedback models hypothesize a change in movement state based on feedforward control (see [3]). In attempting to combine language planning and speech motor control processes, the SFM identifies the phonological level of speech production, where individual speech sounds are encoded into syllable frames, as the level where internal copies of feedforward motor commands are generated. Within such frameworks, interconnected neural networks between the sensory and motor internal models are crucial for error corrections and for training internal feedforward models.

Temporal predictability is the process by which stimuli and sensory events can be anticipated. It occurs when differences between predicted and actual sensory feedback (e.g., auditory) are minimal, thereby allowing for predictability of forthcoming stimuli and events. The Scalar Expectancy Theory (SET [4]) postulates a shared cognitive mechanism across systems that enables temporal expectancy. The SET hypothesized three processes inherent to temporal expectancy: (1) a clock that estimates temporal regularity; (2) a memory state in which memory traces from the present state are compared with a previous state to determine a temporal width comparison; and (3) a decision state.

### 1.2. Effects of Temporal Predictability on Movements

Studies on movement control have provided evidence that tasks that engage temporal expectancy facilitate motor planning, while tasks that engage complexity or response uncertainty have adverse effects [5,6]. Electrophysiological indices of expectancy are the facilitatory effects observed on the premotor *Bereitschaftspotential* and neural oscillatory energy in the beta band (15–30 Hz). Behavioral correlates of expectancy include fewer errors and faster responses [7,8]. For instance, Fujioka et al. [8] investigated beta band oscillations that represent neural oscillatory energy for movement planning. Participants were presented with rhythmic tones—three blocks consisting of fixed tone durations—in comparison to a block of trials consisting of variable tone durations. Magnetoencephalography (MEG) activity showed initial beta band desynchronization that did not correlate with tone rhythm, followed by post-beta synchronization that correlated with tone rhythm. In addition to activity in auditory and parietal cortices, beta synchronization was also associated with neural activity coherence in the motor cortex, including the SMA, pre-SMA, and subcortical regions. The findings were interpreted to suggest a mechanism supporting temporal predictability. Coherence in the absence of motor response was interpreted to suggest sensory and motor neural network coactivation. Using tasks requiring finger movement vs. voice production, Johari and Behroozmand [9] reported comparatively greater suppression of ERP ~100 ms prior to movement onset at frontocentral sites and faster vowel vocalizations and finger movements in a fixed interval than a variable inter-stimulus interval (ISI) condition. Using MEG, Kaltenmaier et al. [10] evaluated participants’ ability to predict timing or spatial orientation of delayed visual probes. Significant rate-specific responses that matched the rhythm of the target were evident in the frontal (SMA), parietal, and temporal regions and correlated with behavioral response in the timing but not the spatial task. Activity in the visual cortex was significantly associated with activity in the motor cortex only in the timing task, thereby suggesting interactions between the sensory and motor cortices in supporting temporal predictability. 

#### Role of Attentional Processes in Temporal Predictability

Endogenous (self-generated in response to cognitive demands; top-down) attentional processes have been reported to occur prior to movement planning in several studies [11,12]. The Contingent Negative Variation (CNV) has been examined as a measure of endogenous attention that enhances sensitivity to temporal predictability in several studies with distributed neural sources from regions that overlap in functions for motor planning and timing. Hillyard [11] demonstrated the association between CNV amplitude from central electrodes and reaction time (RT) in five (sample size = 10) participants in a button-press task to tones using an S1 (cue)-S2 (tone) task with tones that varied in duration. Mento et al. [12] investigated CNV in a S1–S2 audio–visual oddball paradigm that did not require overt responses. CNV amplitude localized to the SMA at the frontocentral and sensory cortical areas and increased with expectation of the standard tone. Coull et al. [13] investigated the neural basis of exogenous (stimulus-based) and endogenous attention in a task engaging temporal decisions. The task required participants to respond to a 50 ms target after a visual cue, which was a valid cue for the target at either a short (600 ms) or long (1400 ms) stimulus-onset asynchrony (SOA). In the invalid cue trials, participants’ attention was drawn to a premature target (exogenous) or required responding to a delayed target (endogenous). RT data showed that participants were significantly faster in the valid trials, suggesting greater temporal predictability. The long SOA in comparison to the short SOA condition showed higher activation in the SMA. Higher activation of the left frontoparietal and bilateral orbitofrontal cortices was evident in the invalid trials engaging attention. 

### 1.3. Purpose of the Study

Temporal predictability or implicit timing is considered to be a relevant phenomenon in the processes supporting speech production. Several studies have provided evidence supporting statistical learning of speech based on temporal regularities in speech input in infants and adult learners (e.g., [14]). Similarly, studies have confirmed a facilitatory effect of temporal regularity in prosody on comprehension (e.g., [15]). Evidence for the facilitatory effects of temporal predictability on speech is also obtained from individuals with neurological impairments. For instance, treatments such as the Melodic Intonation Therapy utilize temporal rhythm to facilitate speech and language production in individuals with Traumatic Brain Injury and aphasia (e.g., [16,17]). Furthermore, stuttering—a fluency disorder—has been attributed to difficulties in rhythm processing, and treatment methods that enhance temporal predictability, including syllable-timed speech and metronome speech, are known to be effective in ameliorating stuttering [18,19]. While suggesting a shared timing mechanism across several cognitive subsystems, such findings motivate the hypothesis that temporal predictability facilitates speech production. However, the extent to which this mechanism influences speech motor processes remains unknown, and the role of implicit attention in enabling temporal predictability effects on speech is yet to be evaluated. Hence, the aim of this study was to investigate if temporal predictability facilitated speech production and the extent to which it influenced speech motor processes and attentional mechanisms supporting such processes. 

We investigated the effects of temporal predictability in an S1 (cue for retrieval)–S2 (cue for impending responses) visually cued delayed-phrase production task. Due to the presence of two visual cues separated by either long (3 s) or short (1.5 s) inter-stimulus intervals (ISIs) and the requirement for delayed overt production, the S1–S2 task allowed the study of both cognitive (implicit attention) and speech motor processes over time and of the effects of temporal predictability on speech production. Neural and behavioral measures of task performance, including ERP and speech onset latencies, respectively, were compared for differences based on ISI. The first question addressed the effects of temporal predictability on the planning and production of phrases in adults at short vs. long ISIs between the S1 and S2 cues. Previous studies of temporal predictability effects on limb movements have used fixed vs. varying ISIs to determine the effects on neural and behavioral responses [7,8,9]. While the speech motor planning and production demands engaged in the short and long ISI trials are similar, the long ISI condition with longer wait time is expected to engage higher temporal expectancy. Based on internal computational models that postulate a feedforward control of speech production (e.g., DIVA, SFM [2,3]), we hypothesized that the long ISI condition with a prolonged wait time will engage neural effort for speech motor planning and production differently than the short ISI condition. Furthermore, based on the SET [4], we hypothesized that the short ISI condition with limited time and resources for engaging relevant cognitive processes (e.g., attentional mechanisms) will show reduced facilitation due to temporal expectancy. As summarized in the introduction, motor decisions involve a combination of processes including temporal expectancy, cortical priming for movements supported by attentional engagement, and response preparedness resulting in action. These motor and cognitive processes have been indexed using ERP in several studies, including the Bereitschaftspotential representing motor planning [20], the CNV representing attentional processes [12,13,21], and the Response-contingent Lateralized Readiness Potential (R-LRP [21]; referred to hereafter as Late Readiness Potential [LRP] representing speech motor programming). Therefore, it was hypothesized that CNV, Bereitschaftspotential, LRP, and speech onset representing the different processes engaged in the S1–S2 visually cued, delayed overt production task would differ between the two ISI conditions. 

In addition to engaging endogenous attention in the task to investigate the effects of temporal predictability on the attentional mechanisms supporting cortical priming for movements, in a second research question, we examined the effects of temporal predictability on cognitive effort through direct experimental manipulation. The S2 visual cue prompted participants to either say the phrase or switch responses to saying the noun rather than the phrase after a temporal delay of 1.5 s. Response switching is considered a lower-level cognitive process that supports higher-level working memory functions. Based on SET [4], interruptions to the process of matching the memory trace of target timing in each trial to the stored representation of target timing will result in interruptions in other associated cognitive processes required for response switching. Hence, it was hypothesized that heightened temporal facilitation in the long ISI condition and lower cognitive interruptions of the temporal memory traces in the speak condition in comparison to the response switch condition will result in enhanced LRP, the ERP index of speech programming, and faster speech onset.

## 2. Materials and Methods

### 2.1. Participants

Twenty-one adults between 18 and 62 years participated in the study (Mean Age: 29 years 3 months, Standard Deviation: 11 years 6 months; 16 females). All participants were right-handed, spoke English as the primary language, and reported absence of hearing, speech, language, attention, or neurological impairments.

### 2.2. Ethics Statement

The study protocol was approved by the Institutional Review Board (STUDY00013042) and was conducted in accordance with the ethical standards outlined in the Declaration of Helsinki. The protocol was approved by the University of Minnesota’s Institutional Review Board. Written informed consent was obtained from all participants in the study.

### 2.3. Materials

Line drawings (7.5 cm × 7 cm) representing familiar monosyllabic nouns were generated. The line drawings varied in the color of the lines to enable participants to name each noun as a phrase. Five colors (white, red, blue, pink, and green) and 11 Consonant-Vowel-Consonant nouns comprised the line drawings. A total of 120 trials were generated with the colors and nouns such that each noun was repeated twice or thrice. Trial order was randomized using a random trial generator and verified further such that identical stimuli were not presented in consecutive trials and trials from the same category occurred no more than twice consecutively.

### 2.4. Experimental Procedure and Design

**Figure 1:**
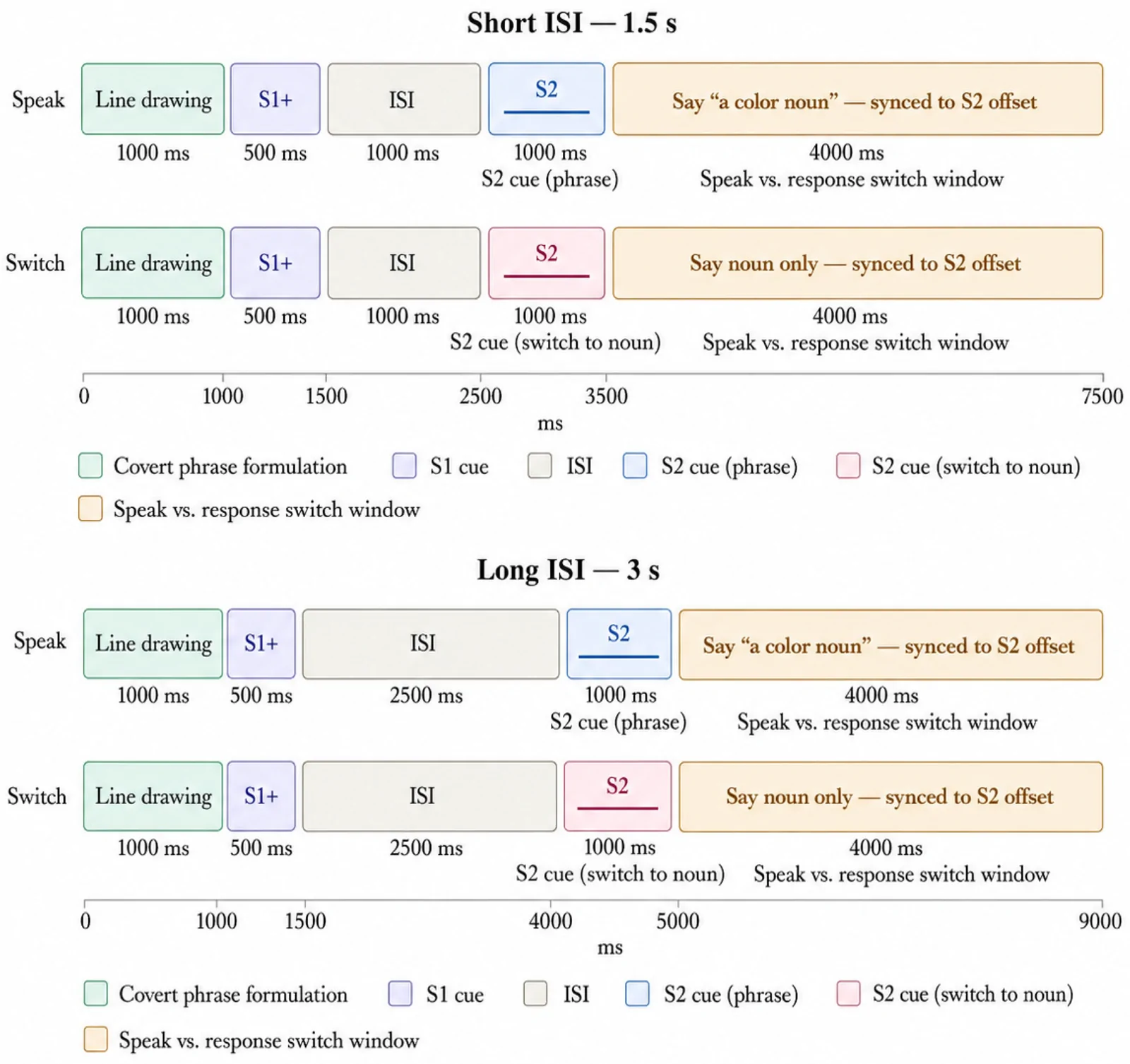
Events in a trial from the short vs. long ISI speak and response switch conditions from the S1–S2 visually cued delayed phrase production task. Image is color-marked for ease of visualization. ISI: inter-stimulus interval.

Testing was conducted in an acoustically and electromagnetically shielded booth at the Center for Translational Sensory Sciences, University of Minnesota. Participants completed three stand-alone tasks in 2 h. The task order was the same across all participants and required participants to respond to the task described in this study first. Mento et al. [12] used an oddball paradigm with trials intermixed for ISI and disengaged ERP markers of cortical priming for movements (that is, CNV) from motor processes. Similarly, in this study, the 1.5 s vs. 3 s ISI trials were pseudorandomized and presented within a set. Figure 1 shows the experimental protocol. Each trial of the S1–S2 visual cue task consisted of the following events. First, participants saw a line drawing that stayed on the screen for 1000 ms. They were required to say the phrase silently during this interval. Second, a crosshair (S1 visual cue; +, 500 ms) appeared on the screen. In the 1.5 s ISI condition (main effect ISI), the crosshair was followed by a second visual cue (S2; “__ ___ ______”) at 1000 ms from S1 offset to prompt the phrase. In the 3 s ISI condition, S1 (500 ms) was followed by the second visual cue (S2) at 2500 ms. In both ISI conditions and in 68% of the trials (speak condition: main effect Cognitive Control), participants were required to say the phrase corresponding to the line drawing (“a color noun”) after a 1000 ms delay when the S2 cue disappeared. In the remaining trials (response switch condition: main effect Cognitive Control), the S2 visual cue was presented within a red rectangle that cued participants to switch to saying only the noun, thereby engaging cognitive control. In all four conditions (2 ISI × 2 Cognitive Control), the last event in a trial stayed on for 4000 ms from S2 offset before moving on to the next trial in the sequence. Participants were familiarized with the nouns before the task, provided with a few practice trials, and instructed to respond to the question “a color WHAT?” in each trial to elicit consistent syllabic stress on the noun. They were instructed to be fast and accurate, to predict the offset of the S2 cue, and to speak approximately in sync with the offset. 

### 2.5. EEG Signal Recording and Analysis

The experimental stimuli were presented using SuperLab Version 6 (Cedrus Inc., Amsterdam, The Netherlands [22]). The EEG data were recorded using a 64-channel BioSemi system (BioSemi V.O.F., Amsterdam, The Netherlands). EEG electrodes were positioned on the scalp according to the standard 10/20 system. EEG data processing was performed using EEGLAB Version 2024 [23]. EEG markers at the onset of the S2 cue from each trial were used to extract ERP responses from the conditions. EEG pre-processing included high- and low-pass filtering between 1 Hz and 40 Hz, 60 Hz line noise filtering using CleanLine Version 2.1 [24], resampling to 512 Hz, and re-referencing to the average of all electrodes. The use of a 1 Hz high-pass filter has been reported to result in ERP amplitude reduction and changes in wave morphology [25]. However, given that the task involved overt speech and was of longer duration, we used a 1 Hz high-pass filter to limit data contamination from movement artifacts and slow drifts. Two methods were used for artifact correction. Independent Component Analysis [23] was used to reject channel noise, eye blinks, movements, and other artifacts from the signal. Using the ICA Picard algorithm [23], spatial components with higher-than-90% probability of being artifacts were rejected. Additionally, extreme movement artifacts were rejected manually by inspecting each EEG file. Data epochs from each trial were extracted between −1000 ms and 3000 ms (S2 cue onset marked 0) for further analysis. Since CNV is expected to occur prior to movement planning, the pre-stimulus interval between −1000 ms and 0 ms was included in the analysis. The duration between −1500 ms and −1000 ms was used for baseline correction. The baseline duration was selected for two reasons. First, at this duration, both ISI conditions are comparable in pre-baseline duration (0 ms to 1000 ms). Second, attentional engagement for motor control processes indexed by CNV is hypothesized to occur later in the task and closer to movement planning [21], and arguably the baseline duration would allow for this component, if present, to be evaluated for a comparable duration (−1000 ms to 0 ms) at both ISIs. Participant responses from the task were recorded using a digital voice recorder and analyzed further using PRAAT [26] to determine accuracy and speech onset latency, which was verified by a trained research assistant to establish reliability. Speech onset latency data from one participant was excluded due to a weak audio signal. Given the proportion of speak vs. response switch trials (0.68:0.32) in the task, there were 637 and 584 trials included in the 3 s and 1.5 s ISI speak conditions, respectively, and 367 trials included in each of the response switch conditions. 

### 2.6. Statistical Analysis

ERP activity from the premotor and motor cortices and from the parietal cortex is reported given the roles attributed to these regions in sensorimotor control. Furthermore, the anterior frontal electrodes were analyzed by hemisphere due to the possibly varying roles of the left vs. right hemispheres in speech production and cognitive control. The anterior frontal electrodes included average ERP from left vs. right frontal pole, anterior frontal, and frontal electrodes (left and midline anterior frontal electrodes: Fp1, AF7, AF3, F1, F3, F5, F7, and Fpz; right anterior frontal electrodes: Fp2, AF4, AF8, F2, F4, F6, F8) representing the left and right frontal pole and the superior, medial, and inferior frontal gyri [27]. Additionally, the average ERP from bilateral central electrodes (C1, C2, C3, C4, C5, C6, Cz) was included in the analysis. Bilateral parietal electrodes (P1, P2, P3, P4, P5, P5, P7, P8, and Pz) included cortical regions representing the precuneus, superior parietal, and lateral occipital regions [27]. Given the role attributed to attentional mechanisms in movement control [4,12,13,21], CNV was included as a covariate in the analysis. At each latency of interest, linear mixed effects analysis (lmer, R package Version 2.0-6 [28]) was conducted on the mean amplitude between latencies of interest, with ISI (1.5 s vs. 3 s) and Cognitive Control (speak vs. response switch) as categorical independent variables and participants as random effects. When heteroscedasticity of variances was observed (Breusch Pagan test, *p* < 0.05), robust random error estimates (rlmer; R package Version 3.4-2 [29]) were used to limit the influences of extreme values. Satterthwaite’s degrees of freedom (df) adjustments were used in both analyses. Posthoc comparisons were conducted using Tukey’s Highly Significant Difference estimates (emmeans package Version 2.0.3 [30]). Statistical output including F and *p*-values is reported for linear mixed effects analysis; t values, *p*-values, and confidence intervals (CI) are reported for robust mixed effects analysis. Given the several variables included in the analyses, post hoc comparisons were limited to main effects and two-way interactions. Very few errors were noted across participants, and these were evenly distributed across categories (Mean Accuracy = 97.8%, SD = 1.9). Statistical analyses were conducted using EEGLAB Version 2024, ERPLAB Version 12 [31] and R Version 4.4.1 (R Core Team [32]; see Code S1 and Table S1 in Supplemental Materials for Sample R code and data from anterior frontal electrodes and RT data). Speech onset latency (ms), defined as the duration from the offset of the S2 cue to the onset of participants’ speech in each trial, was obtained using PRAAT and analyzed using linear mixed effects (Breusch Pagan test, *p* > 0.05), with ISI and Cognitive Control as independent variables. 

## 3. Results

### 3.1. ERP Data

**Figure 2:**
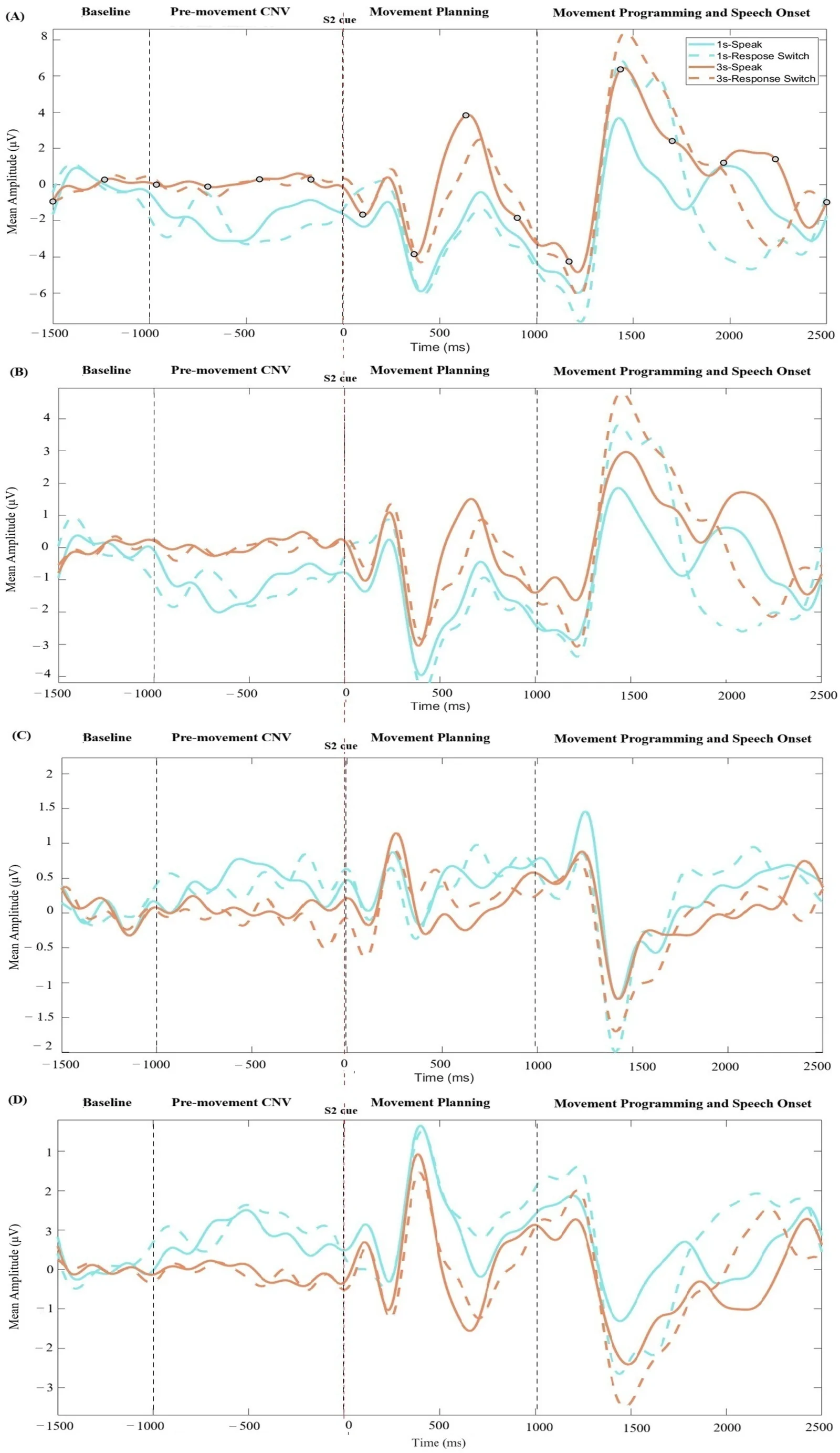
Mean amplitude and time course of ERP between latencies −600 ms and −400 ms (pre-movement CNV), 600 and 400 ms (movement planning, Bereitschaftspotential), and 2000 and 2300 ms (movement programming, LRP) with reference to S2 cue onset (0 ms) at: (**A**) left anterior frontal (average of Fp1, Fpz, AF3, AF7, Fz, F1, F3, F5, F7); (**B**) right anterior frontal (average of Fp2, AF4, AF8, F2, F4, F6, F8); (**C**) bilateral central (average of Cz, C1, C2, C3, C4, C5, C6); and (**D**) bilateral parietal (average of Pz, P1, P2, P3, P4, P5, P6, P7, P8) electrodes. Comparisons are shown for 1.5 s (short) vs. 3 s (long) ISI by speak vs. response switch requirements in the S1–S2 delayed overt production task. CNV: Contingent Negative Variation; LRP: Late Readiness Potential.

Initial analysis of the ERP data showed significant omnibus effects at three latencies with reference to the S2 cue onset (−600 ms to −400 ms, 2000 to 2300 ms at anterior frontal and parietal electrodes; 400 ms to 600 ms at anterior frontal electrodes; *p*-values ≤ 0.05). The central electrodes were also included in the analysis for comparison. Figure 2 shows ERP mean amplitudes from the left vs. right anterior frontal, bilateral central and parietal electrodes at the latencies.

#### 3.1.1. Latency −600 ms to −400 ms

The first significant effect occurred between −600 ms and −400 ms prior to S2 onset, when CNV, the marker of attentional effort, was expected to occur. A comparison of mean amplitude from the anterior frontal electrodes by ISI, Cognitive Control, and Hemisphere showed a significant main effect of ISI, F(1,144) = 13.15, *p* < 0.001. A post hoc comparison showed a significantly larger CNV response in the 1.5 s condition (Mean = −13.9, SE = 3.5, lower CI = −21.1, upper CI = −6.8) in comparison to the 3 s ISI condition (Mean = 4.6, SE = 2.1, lower CI = −2.4, upper CI = 11.8) immediately prior to S2 cue onset. All other main and interaction effects were non-significant. 

A comparison of CNV at bilateral central electrodes showed a significant main effect of ISI, F(1,60) = 10.9, *p* = 0.001, and a significant interaction effect of ISI × Cognitive Control, F(1,60) = 5.4, *p* = 0.023. The main effect of ISI resulted in a larger CNV mean amplitude in the 1.5 s ISI (Mean = 3.3, lower CI = 1.5, upper CI = 5.2) in comparison to the 3 s ISI condition (Mean = −0.42.1, lower CI = −2.2, upper CI = 1.4). The interaction effect showed that CNV amplitude was significantly larger in the 1.5 s ISI condition that required overt speech rather than response switching (*p* = 0.003). All other comparisons were non-significant (see Table 1). Comparison of CNV at bilateral parietal electrodes resulted in a significant main effect of ISI, F(1,60) = 6.6, *p* = 0.01, with larger CNV amplitude in the 1.5 s ISI (Mean = 8.2, lower CI = 2.4, upper CI = 14.1) in comparison to the 3 s ISI condition (Mean = −2.3, lower CI = −8.2, upper CI = 3.4).

#### 3.1.2. Latency 400 ms to 600 ms

**Figure 3:**
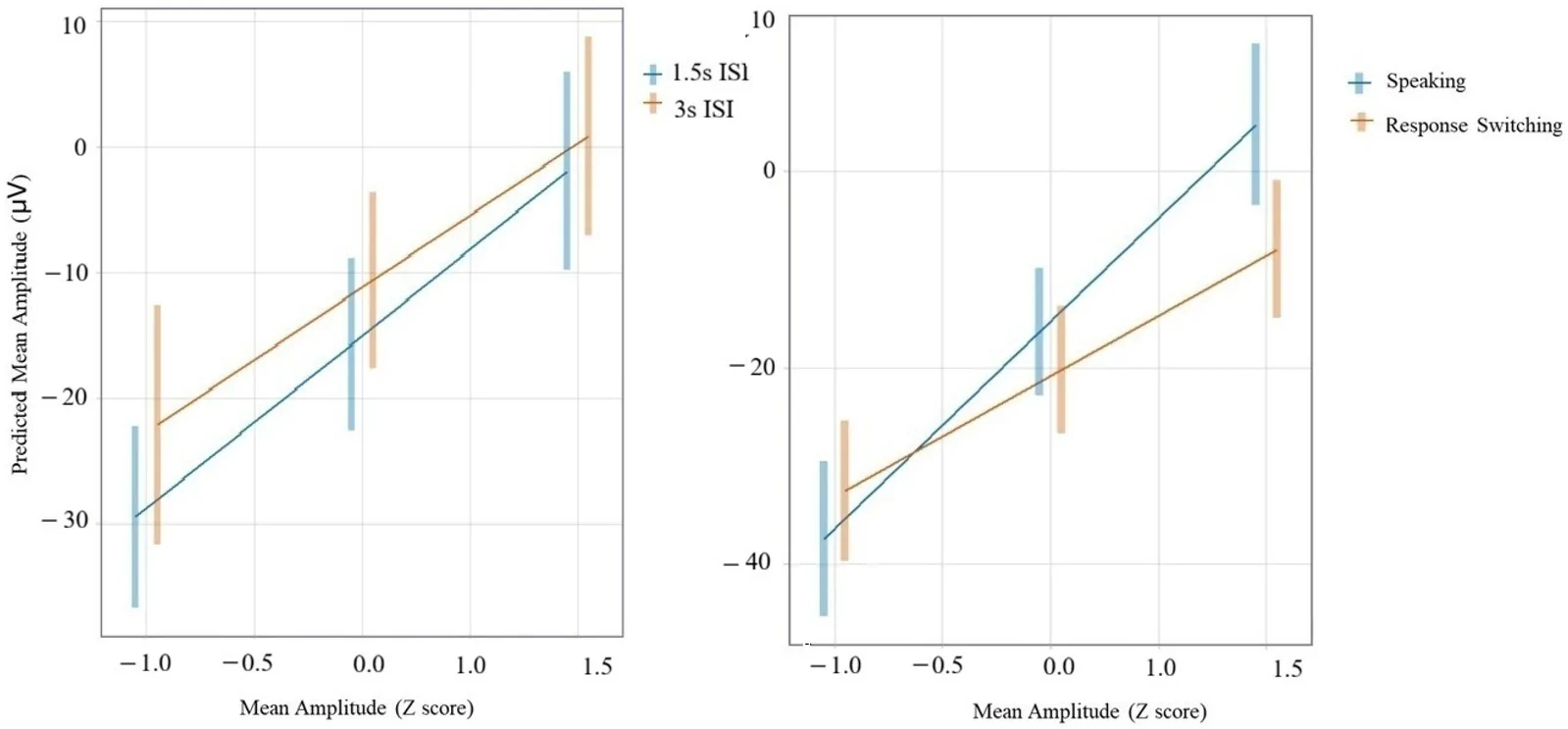
Mean amplitude and CI of Bereitschaftspotential (y-axis) at bilateral anterior frontal electrodes by ISI (1.5 s vs. 3 s; (**left**) panel) and Cognitive Control requirements (speak vs. response switch; (**right**) panel) modulated by mean amplitude of Contingent Negative Variation as covariate (x-axis). ISI: inter-stimulus interval; CI: confidence interval.

The second significant effect occurred between 400 ms and 600 ms at the anterior frontal electrodes when the Bereitschaftspotential, the ERP marker of motor planning, was expected to occur. ISI, Cognitive Control, and Hemisphere as main effects and mean amplitude of CNV (Z-transformed scores) from the anterior frontal electrodes resulted in significant main effects of ISI (1.5 s ISI > 3 s ISI), t(1,140) = 2.6, *p* = 0.009, and Cognitive Control (response switch > speak condition), t(1,138) = −2.8, *p* = 0.004 (see Table 2). The covariate effect of CNV, t(1,150) = 6.1, *p* < 0.001, and the interaction effects of ISI × CNV, t(1,146) = −2.3, *p* = 0.017, and Cognitive Control × CNV, t(1,153)= −2.8, *p* = 0.006, were also significant (see Figure 3). Variations in CNV amplitude influenced the mean amplitude of the Bereitschaftspotential in the 1.5 s ISI condition to a greater extent, and the differences between the conditions were significant at the mean value of CNV (simple slopes test: CNV Z score = 0; Z ratio = −3.9, *p* < 0.001). Similarly, variations in CNV influenced the speak condition to a greater extent, and the differences between the speak vs. response switch conditions were significant at higher-than-mean CNV values (CNV Z score = 0; Z ratio = 3.4, *p* < 0.001). A similar interaction of ISI × CNV was also evident at bilateral central electrodes, F(1,63) = 4.6, *p* = 0.034.

Analysis at bilateral parietal electrodes resulted in a main effect of Cognitive Control, t(1,58) = 2.1, *p* = 0.035, with a larger deflection of Bereitschaftspotential in the response switch condition (Mean = 4.5, lower CI = 1.4, upper CI = 7.7) in comparison to the speak condition (Mean = 2.3, lower CI = −0.79, upper CI = 5.5). The covariate effect of CNV was also significant, t(1,70) = 2.2, *p* = 0.034.

#### 3.1.3. Latency 2000 ms to 2300 ms

Significant differences were observed at latencies of 2000 ms to 2300 ms when the ERP associated with movement programming, LRP, was expected to occur. Robust mixed effects analysis of LRP mean amplitude from the anterior frontal electrode sites was conducted with ISI, Cognitive Control, and Hemisphere as independent categorical variables and CNV (Z scores) as a covariate. This analysis resulted in a significant main effect of Cognitive Control, t(1,138) = −4.9, *p* < 0.001. The response switch condition (Mean = −21.3, lower CI = −27.2, upper CI = −15.5) showed significantly larger LRP deflection at this latency compared to the speak condition (Mean = 4.8, lower CI = −1.06, upper CI = 10.7). A significant covariate effect of CNV was also obtained, t(1,156) = 4.4, *p* < 0.001. The interaction of Cognitive Control × Hemisphere showed a trend towards significance t(1,136) = 1.8, *p* = 0.06. Similarly, the interaction effect of Cognitive Control × CNV also showed a trend towards significance, t = −1.9, *p* = 0.054.

Analysis of bilateral central electrodes revealed a significant covariate effect of CNV, F(1,77) = 4.9, *p* < 0.03. Analysis of the parietal electrodes showed a significant main effect of Cognitive Control, F(1,77) = 17.5, *p* = 0.007. A larger ERP deflection was evident in the response switch (Mean = 13.9, lower CI = 7.48, upper CI = 20.47) in comparison to the speak condition (Mean = −3.1, SE = 3.2, lower CI = −9.6, upper CI = 3.8). The covariate effect of CNV was also significant, F(1,76) = 16.8, *p* < 0.001. All other main and interaction effects were non-significant.

### 3.2. Behavioral Data

**Figure 4:**
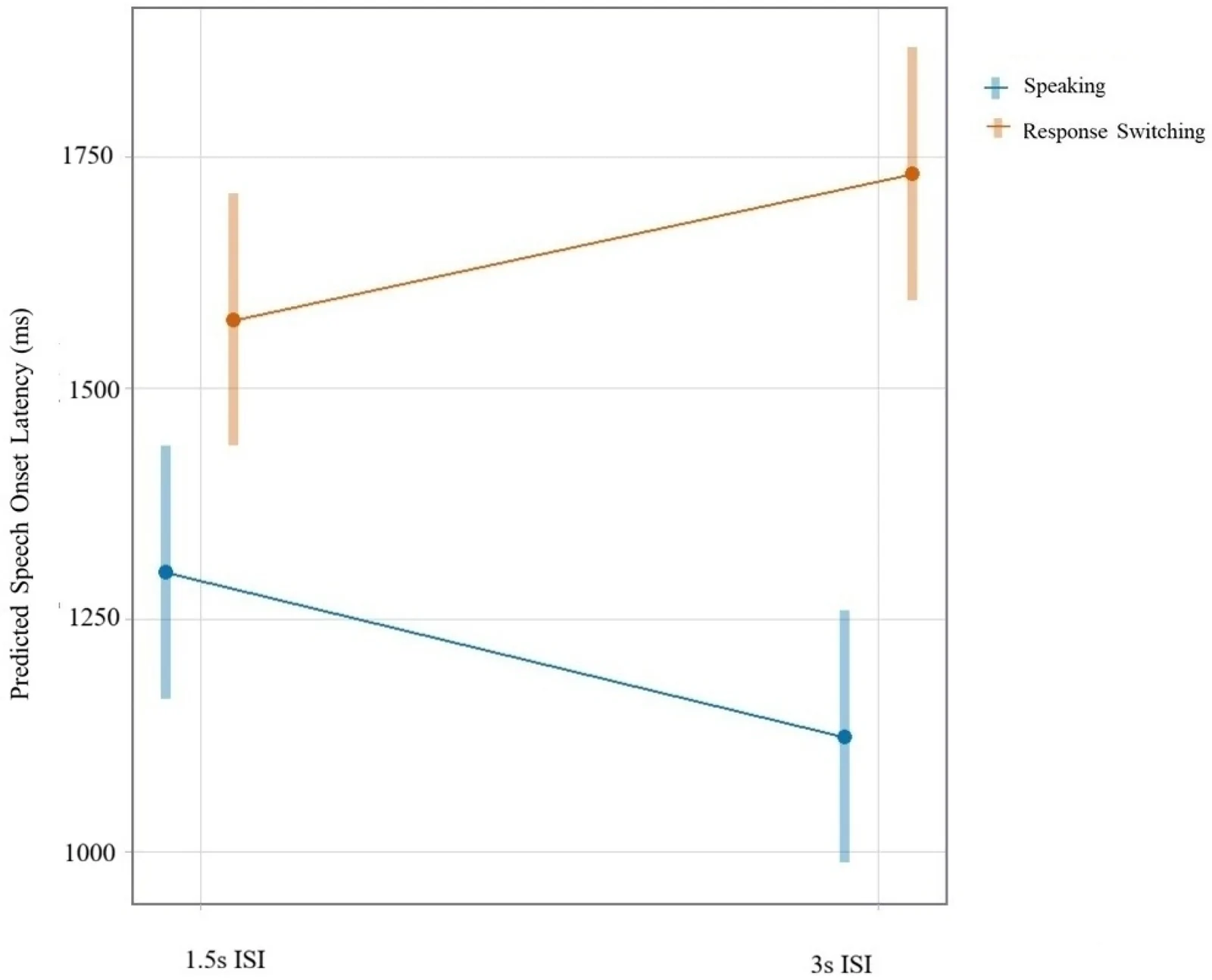
Average speech onset latency (ms) and confidence interval by ISI (1.5 s vs. 3 s) and Cognitive Control (speaking vs. response switching).

The RT data were analyzed to determine if the conditions differed in speech onset latencies. CNV (from left anterior frontal electrodes) was included as a covariate in the analysis. The main effect of Cognitive Control was significant, F (1,57) = 55.3, *p* < 0.0001. Average speech onset latency in the speak condition (Mean = 1212, SE = 49.1) was significantly faster in comparison to the response switch condition (Mean = 1652, SE = 49.1; t = −6.3, *p* < 0.001). A significant interaction effect of ISI × Cognitive Control was obtained, t (1,57) = 7.8, *p* = 0.008 (see Figure 4). Post hoc comparisons showed significantly slower average speech onset latency in the response switch trials at both ISIs in comparison to the speak trials (1.5 s response switch vs. speak, *p* = 0.02, 3 s response switch vs. 3 s speak, *p* < 0.0001; 3 s response switch vs. 1.5 s speak, *p* = 0.0001; 1.5 s response switch vs. 3 s speak, *p* = 0.0001). Responses to the 3 s response switch condition were significantly slower in comparison to the other conditions. All other comparisons were non-significant.

## 4. Discussion

This study investigated the effects of temporal predictability on speech motor processes in a delayed overt phrase production task. ERP markers of processes that are influenced by intrinsic temporal expectancy, including speech motor processes and the supporting cognitive processes, were investigated. Additionally, we investigated whether temporal expectancy influenced cognitive effort associated with response switching. Speech onset latencies were compared between conditions and interpreted in conjunction with ERP. 

In response to the first research question, significant differences in mean amplitude (1.5 s > 3 s) were evident in CNV ~500 ms prior to S2 cue onset, suggesting differences between the ISI conditions in cortical priming for attentional engagement prior to movement planning observed at the anterior frontal, central, and parietal electrode sites. At the central electrodes, CNV amplitude varied by ISI in the speak but not in the response switch condition. Differences (1.5 s > 3 s) were observed in the Bereitschaftspotential between 400 and 600 ms post-S2 cue onset and suggested that speech motor planning differed between the short vs. long ISI conditions by temporal predictability. However, in spite of comparable temporal expectancy and amplitude of the Bereitschaftspotential responses, the 3 s response switch condition showed significantly slower speech onset latency in comparison to the 3 s speak condition and was comparable to the 1.5 s response switch condition in LRP, a measure of speech programming. This suggested that rather than temporal expectancy, which would have resulted in ISI-based differences, demands on cognitive control and response switching influenced LRP. The findings are interpreted chronometrically with reference to S2 cue onset and in terms of the processes supporting temporal expectancy.

### 4.1. Temporal Predictability and Attentional Engagement

Significant differences by ISI were evident between 400 ms and 600 ms prior to S2 onset. This ERP response occurred at bilateral anterior frontal, central, and parietal electrodes at the latency at which the CNV was expected to occur. The occurrence of CNV is suggested to be a signal of cortical priming for movement planning [11,21]. Therefore, given the higher temporal expectancy in the long ISI condition, differences were expected between the ISI conditions and were confirmed. Interestingly, it was the short ISI condition that showed the most deflection in CNV amplitude at the time point that immediately preceded the S2. The absence of a similar CNV effect in the long ISI condition is attributed to the prolonged wait duration (an additional 1 s beyond the S1 cue) that may have resulted in early temporal decisions across trials at latencies immediately after S1. The observed differences at short ISI confirmed the hypothesis postulated by SET [4] that attributes an accruing threshold for temporal prediction until the point of S2 onset. In the long ISI condition, decision on the temporal structure of an interval would have already occurred when the ISI duration exceeded 1.5 s. In the short ISI condition, this decision is expected to occur immediately prior to S2 onset when attentional and working memory processes are actively engaged in evaluating temporal similarity between trials. Hence, larger CNV deflection in the short ISI condition is interpreted to represent ongoing temporal decision. It could also represent trial-by-trial variability in the decisions on temporal structure that can be expected in the short ISI trials. This was confirmed through the higher standard deviation observed in the 1.5 s response switch condition, which engaged higher cognitive effort. These findings are supported by the findings and interpretation from Hillyard [11] that the intention to respond at a precise time rather than response timing influenced the amplitude of the CNV. The interaction between ISI and Cognitive Control at the central electrodes suggested that in addition to differences based on ISI, attentional effort was also influenced by the requirements for speaking over response switching, suggesting heightened attentional salience to trials requiring immediate response (that is, the 3 s speak condition). On a cautionary note, attentional salience has also been attributed a relevant role in movement control [33]. Based on a review, Heinrich et al. [33] attributed attentional effort and impulsive responses to the left prefrontal cortex vs. alertness and slower responses to the right prefrontal cortex. The findings suggested that attentional effort was most engaged by trials that required additional resources due to the requirements for just-in-time processing (e.g., short ISI trials, speak trials). Future studies can disambiguate attentional salience vs. effort for their roles in movement control by evaluating CNV differences by hemisphere, particularly since, rather than confirming attentional salience, the lack of attentional effort had to be inferred in the long ISI speak condition. The CNV was evident at anterior frontal, central, and parietal electrodes, and confirmed similar findings in other studies. While structures in the anterior frontal cortical regions (e.g., SMA) are attributed to temporal expectancy and cognitive control, the parietal region is attributed to stimulus-driven attentional engagement [12,13]. Both intrinsic temporal expectancy and cortical priming for movements (the latter indexed by CNV) and extrinsic attentional engagement were elicited in this study through the use of ISI-based implicit cueing and visual cues for speaking vs. response switching, respectively. 

### 4.2. Temporal Predictability and Speech Motor Processes

In response to the first research question, the findings confirmed varying effects of temporal predictability on speech motor planning between the long vs. short ISI conditions, which were localized to the bilateral anterior frontal regions [34]. A Steeper ERP deflection was observed in the 3 s ISI condition between 400 ms and 600 ms post-S2 onset. This is interpreted to suggest that decision processes associated with movement planning were achieved earlier in this condition, whereas they were prolonged in the 1.5 s ISI condition. The Bereitschaftspotential has been attributed to motor planning and is influenced by task-related processes including predictability and/or uncertainty. This interpretation is also supported by the findings from CNV that suggested ongoing engagement of cognitive mechanisms supporting temporal decisions in the short ISI condition at S2 onset. This could have resulted in competing cognitive resources, thereby creating sequential delays in speech motor planning. Accordingly, the effect of CNV on the Bereitschaftspotential was larger in the short ISI trials. Additionally, the CNV was larger in the speak condition than in the response switch condition. Together, these findings suggested that immediately prior to the cue for overt speech, attentional resources were prioritized to trials requiring ongoing processing. These findings may provide support for feedforward accounts of speech motor control [2,3] by suggesting that in an overt production task involving higher accuracy and a limited role for feedback, temporal predictability may be represented within the feedforward signal and could influence motor planning. 

Significant differences were evident between the long vs. short ISI speak conditions in attention-mediated speech motor planning. This finding suggested that the speech motor system is sensitive to inherent differences in rhythmicity, with slower temporal rhythm exerting a greater facilitatory effect on speech motor planning (e.g., [35]). This interpretation is supported by Mento et al. [12], who found SMA activation in long trials in comparison to short SOA trials in an S1–S2 audio–visual cue task. However, the speak trials at both ISIs were comparable in LRP, and average speech onset latency in the speak trials was significantly faster in comparison to the response switch trials. While seemingly contradictory, an interpretation is that the effect of temporal predictability is limited to movement planning and does not extend to movement preparation and speech onset when the task involves delayed temporal decisions that limit predictability (e.g., short ISI). Another, more plausible interpretation is that earlier response decisions and the requirement for delayed overt production allowed some participants to adopt individualized speech onset strategies, with some initiating speech before S2 cue offset in the long ISI speak trials. Nevertheless, the long ISI speak condition showed facilitation at all levels, including attentional salience (by the absence of CNV immediately prior to movement planning), movement planning, programming, and speech onset.

It was expected that the facilitatory effect of temporal expectancy would extend to LRP and speech onset as a carry-over effect from speech motor planning. Instead, a significant main effect of Cognitive Control was evident in both LRP and speech onset latency. Studies of LRP have suggested that pre-cueing influences LRP amplitude [36]. In this study, the pre-cue for both speaking and response switching was the S2 visual cue. Hence, we argue that the inherent cognitive complexity associated with response switching, together with the unequal proportion of speak vs. response switch trials (0.68:0.32), resulted in the observed differences. Perhaps, the strong effect of response switching on LRP could also partially account for the diminished effect of ISI on LRP. This effect was localized to both anterior frontal and parietal electrodes, suggesting a higher-level cognitive component and a visual component, which conformed with the task requirements. 

In response to the second research question, two findings are used to infer cognitive control mechanisms supporting speech planning, production, and response switching. First, ISI-based differences in CNV evident in the bilateral anterior frontal, central, and parietal regions supported the interpretation that cortical priming for movements that is influenced by temporal expectancy is a domain-general mechanism with multiple neural sources. Furthermore, ERP responses specific to speech motor planning occurred in the anterior frontal and central cortical regions, while response switching was localized to the bilateral anterior frontal and parietal cortical regions. These findings conveyed varying roles for these cortical regions in encoding cues for attentional salience, speech motor planning, and response switching. The dorso-lateral prefrontal cortex is part of the frontoparietal network attributed to domain-general task-set reconfiguration, attention, and goal-directed behavior [36,37], while the SMA is attributed to encoding of temporal rhythmicity [8,10]. Neural pathways connecting these regions with the parietal and occipital cortices influence sensory processing through precise encoding of task-specific rules and stimulus information. Second, significant differences in LRP and slower onset latencies in the response switch condition suggested higher response complexity associated with response switching (e.g., [37]). Furthermore, differences associated with the requirements for response switching vs. speaking were evident in the parietal region between 400 ms and 600 ms and in the anterior frontal and parietal regions between 2000 ms and 2300 ms. Given that response switching has been attributed to cortical control mechanisms engaging the prefrontal, bilateral parietal and occipital regions [38], the effort associated with response switching is likely to have a cortical source. Although articulatory complexity could also account for such differences, response switching in the task involved reduced articulatory complexity (saying monosyllables instead of trisyllables). Therefore, rather than peripheral motor complexity, which could have also accounted for the observed effects of cognitive control on LRP and speech onset latency, the requirements for response set switching are attributed to the delay observed in the response switch condition. Hence, similarities and differences between speaking vs. response switching by ISI suggested varying effects of temporal predictability on speech motor planning and production. The oddball task design necessitated fewer response switch trials; future studies can compare a proportionate number of speak and response switch trials to further validate the differences observed in this study.

## 5. Conclusions

This study identified ERP markers sensitive to the facilitatory effects of temporal predictability on speech production and provided evidence supporting such effects, which can be broadly addressed within feedforward models of speech [2,3] and models of temporal predictability [4]. The findings also provide preliminary evidence that ISI effects are shared across both limb and speech motor domains. This could suggest shared neural substrates supporting processes involved in temporal expectancy. This is particularly relevant in the context of rehabilitation strategies for speech motor disorders that use both speech and non-speech movements to facilitate motor responses.

A few limitations are noted. First, we did not observe advanced cortical priming for movement (CNV) in the long ISI condition, which could be due to the baseline duration selected to compare the conditions. Such an effect would confirm hypotheses based on the SET at both ISIs. At short ISI, motor planning was prolonged and is attributed to ongoing decision-making on temporal structure supported by attentional engagement. Differences in the Bereitschaftspotential confirmed a facilitatory effect of temporal expectancy on speech motor planning at longer ISIs and slower temporal rhythm, which was also evident in speech onset latency. However, similar facilitation was not evident in LRP and speech onset latency, thereby suggesting that while temporal predictability enables attention-facilitated speech motor planning and in some instances faster speech onset, other factors influence motor programming for speech onset, thereby limiting the effects of temporal predictability. Furthermore, the findings are tentative to the extent that ERP can be used to interpret cortical mechanisms (e.g., temporal expectancy) and require further testing using imaging methods to localize the observed differences to cortical centers attributed to such mechanisms (e.g., SMA).

## Figures and Tables

**Table 1:** Mean amplitude and 95% CI of Contingent Negative Variation (CNV) by ISI (1.5 s vs. 3 s) and Cognitive Control (speak vs. response switch) at bilateral central electrodes. ISI: inter-stimulus interval; CI: confidence interval.

ISI	Cognitive Control	Mean Amplitude (μV)	Lower CI	Upper CI
1.5 s	Speak	4.6	2.1	7.2
1.5 s	Response Switch	2.1	−0.5	4.6
3 s	Speak	−1.4	−4.3	0.7
3 s	Response Switch	−0.9	−1.6	3.5

**Table 2:** Mean amplitude and CI of Bereitschaftspotential by ISI (1.5 s vs. 3 s) and Cognitive Control (speak vs. response switch) main effects at anterior frontal electrodes. ISI: inter-stimulus interval; CI: confidence interval.

Main Effect	Condition Contrast	Mean	95% CI (Lower)	95% CI (Upper)	Statistical Significance (*p*)
ISI	1.5 s ISI	−20.7	−27.6	−13.7	0.009
	3 s ISI	−10.9	−17.8	−3.8	
Cognitive Control	Response Switch	−20.1	−26.9	−13.2	0.004
	Speak	−11.4	−18.3	−4.5	

## Data Availability

The raw data supporting the conclusions of this article can be retrieved from: https://doi.org/10.13020/r488-8v09.

## Supplementary Materials

The following supporting information can be downloaded at: https://www.mdpi.com/article/10.3390/brainsci16080817/s1, R Code S1: Sample R code for data analysis; Table S1: ERP data from the anterior frontal electrodes between 400 ms and 600 ms, and RT data are provided.

## Abbreviations

The following abbreviations are used in this manuscript:

ERP	Event-Related Potential
ISI	Inter-Stimulus Interval
CNV	Contingent Negative Variation
LRP	Response-contingent Lateralized Readiness Potential
SET	Scalar Expectancy Theory
